# Degenerative Zygapophyseal Joint Subluxation of the Lumbar Spine: A Modified Classification and Clinical Considerations

**DOI:** 10.7759/cureus.99484

**Published:** 2025-12-17

**Authors:** Stylianos Kapetanakis, Mikail Chatzivasiliadis, Krikor Gkoumousian, Constantinos Chaniotakis

**Affiliations:** 1 Spine Department and Deformities, Interbalkan European Medical Center, Thessaloniki, GRC

**Keywords:** dislocation, facet joint, lateral recess stenosis, subluxation, zygapophyseal joint

## Abstract

Degenerative lumbar zygapophyseal (facet) joint subluxation without prior trauma, fracture, spondylolysis, or spondylolisthesis is extremely uncommon and represents a distinct clinical entity. This study aims to present rare cases of advanced degenerative facet joint changes combined with unilateral or bilateral facet joint subluxation in the absence of trauma. All patients experienced chronic symptoms unresponsive to conservative treatment. MRI findings demonstrated Grade 4 degeneration per Grogan’s classification, along with distinct subluxation patterns not captured by existing grading systems. Surgical treatment, including decompression with or without instrumented fusion, was tailored to the presence of radiological or intraoperative instability. These findings emphasize the clinical relevance of facet joint subluxation as a distinct degenerative entity, suggesting the need for a revised MRI-based grading system that includes subluxation as a separate criterion. This revised classification system, incorporating degenerative causes, could enhance diagnostic precision and therapeutic strategies in these types of injuries.

## Introduction

Degenerative changes of the zygapophyseal joint (facet joint), known as facet joint osteoarthritis (FJO), are increasingly common with age, affecting more than 50% of the elderly population [1,2]. FJO is strongly associated with degeneration of the intervertebral discs and instability in the lumbar spine, and it plays a significant role in the onset and progression of lumbar foraminal stenosis [3-5]. Grogan et al. created an MRI grading system that can be used to assess degenerative changes in facet joints [6]. It classifies the severity and extent of cartilage loss, subchondral bone alterations, and osteophyte formation into four separate grades [6]. However, Grogan’s system does not currently account for cases where degeneration could potentially lead to mechanical instabilities like facet joint subluxation.

Lumbar facet joint subluxation or dislocation is a rare condition, unlike the more commonly reported facet joint subluxations of the cervical spine [3,4]. Most reported cases of facet joint dislocations are associated with high-energy trauma, such as motor vehicle accidents or high falls which involve a distractive mechanism combined with flexion of the lumbar spine [4,5]. Additionally, these traumatic cases are almost always accompanied by fractures. On the contrary, facet joint subluxation (unilateral or bilateral) is a finding commonly observed in cases of degenerative spondylolisthesis in the lumbar spine [4].

In our study, we present a case series of patients with degenerative changes of the lumbar facet joints that are of particular clinical interest, combined with a rare pathology of degenerative facet joint subluxation in the absence of trauma, fractures, or spondylolisthesis. While degenerative changes in the lumbar spine are common causes of lumbar stenosis, the presence of facet subluxation without vertebral displacement is unique. To our knowledge, these are the first reported cases of degenerative facet joint subluxation in the lumbar spine in combination with degenerative changes, making it a unique contribution to the literature. We would also like to note that there is no classification system for magnetic resonance imaging (MRI) findings of the cases present in the existing literature. For this reason, we propose modifying the Grogan classification so that cases similar to ours are classified as Grade V [6].

## Case presentation

We retrospectively reviewed lumbar spine MRI of elderly patients (>65 years old) who presented to our outpatient clinic with low back pain, with or without leg pain (either unilateral or bilateral) [7]. From this cohort, we selected cases that demonstrated notable degenerative changes of the facet joints. The degree of facet joint degeneration was classified using the Grogan grading system [6,8]. In Stage I, the articular processes are lined by thin cortical bone, and the joint space appears as a uniform dark band of low MR signal. In Stage II, focal thickening of the cortical bone develops, with cartilage still covering the surface but showing areas of erosion or irregularity, resulting in an uneven appearance of the interspace. In Stage III, cortical thickening involves less than half of the articular surface, cartilage coverage becomes incomplete, and portions of the underlying bone are exposed within the joint space. Finally, in Stage IV, dense cortical bone covers more than half of the surface with osteophyte formation; cartilage is almost completely absent, leaving only remnants and low-signal voids visible within the interspace [6,8]. All authors jointly reviewed the MRIs to ensure consensus in the selection of representative cases. Clinical information and MRI findings of the included cases are presented in detail. Written informed consent to participate in our study was obtained from all patients.

Case 1

**Figure 1:**
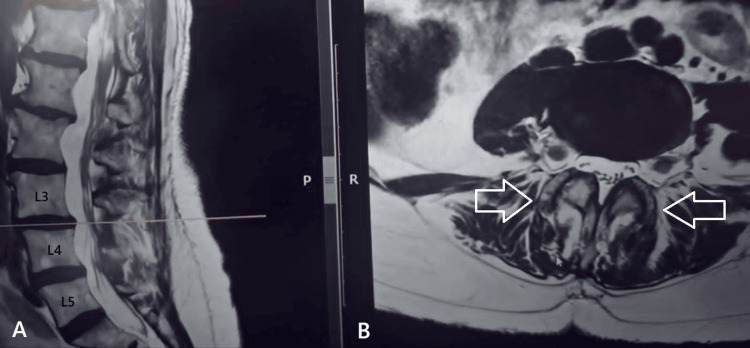
(A) T2-sagittal view and (B) T2-axial MRI views of the lumbar spine in first case. At the L3-L4 level, the arrows with the white outline indicate the hypertrophic facets (Grade 4 according to Grogan) with accompanying bilateral facet subluxation.

The first case involved a 70-year-old woman with a two-year history of low back pain, which had progressively worsened over the past three months and was accompanied by bilateral leg pain and severe neurogenic claudication (approximately 100 meters of walking distance). Clinical examination revealed bilateral muscle strength graded at 4/5, sensory deficits, and normal tendon reflexes in the L3-L4 distribution. Lumbar spine MRI showed central canal stenosis with Grade 4 degenerative changes of the facet joints according to the Grogan classification, along with bilateral facet joint subluxation at the L3-L4 level (Fig. 1). The patient underwent posterior decompression and posterolateral instrumented fusion at L3-L4 using transpedicular screws and rods. She experienced immediate postoperative relief, was mobilized six hours after surgery, and was discharged the following day with physiotherapy instructions.

Case 2

**Figure 2:**
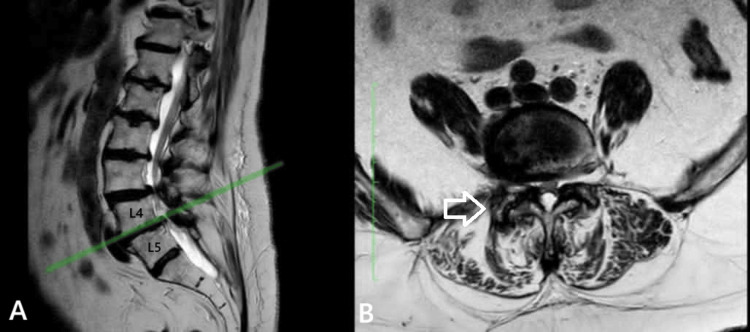
(A) T2-sagittal view and (B) T2-axial MRI views of the lumbar spine in second case. At the L4-L5 level, the arrows outlined in white denote the right-sided hypertrophic facet joint (grade 4 according to Grogan), exhibiting a tendency toward facet subluxation.

The second case involved a 68-year-old woman with a one-year history of low back pain and progressive right-sided sciatica over the past two months. On examination, she demonstrated right lower limb muscle strength of 4/5, accompanied by hypoesthesia and normal tendon reflexes in the right L4 distribution. Lumbar spine MRI revealed right-sided lateral recess and foraminal stenosis at the L4-L5 level, with Grade 4 degenerative changes of the facet joint according to the Grogan classification (Fig. 2). Notably, the right facet joint showed signs of impending subluxation, while degenerative changes were also present in the left facet joint but without clinical symptoms. The patient underwent a right-sided semilaminectomy at L4-L5, foraminotomy, and partial (40%) facet joint resection to avoid instability. Intraoperatively, no instability was observed following decompression. The patient experienced immediate postoperative symptom relief, was mobilized six hours after surgery, and was discharged 12 hours later under a physiotherapy rehabilitation protocol.

Case 3

**Figure 3:**
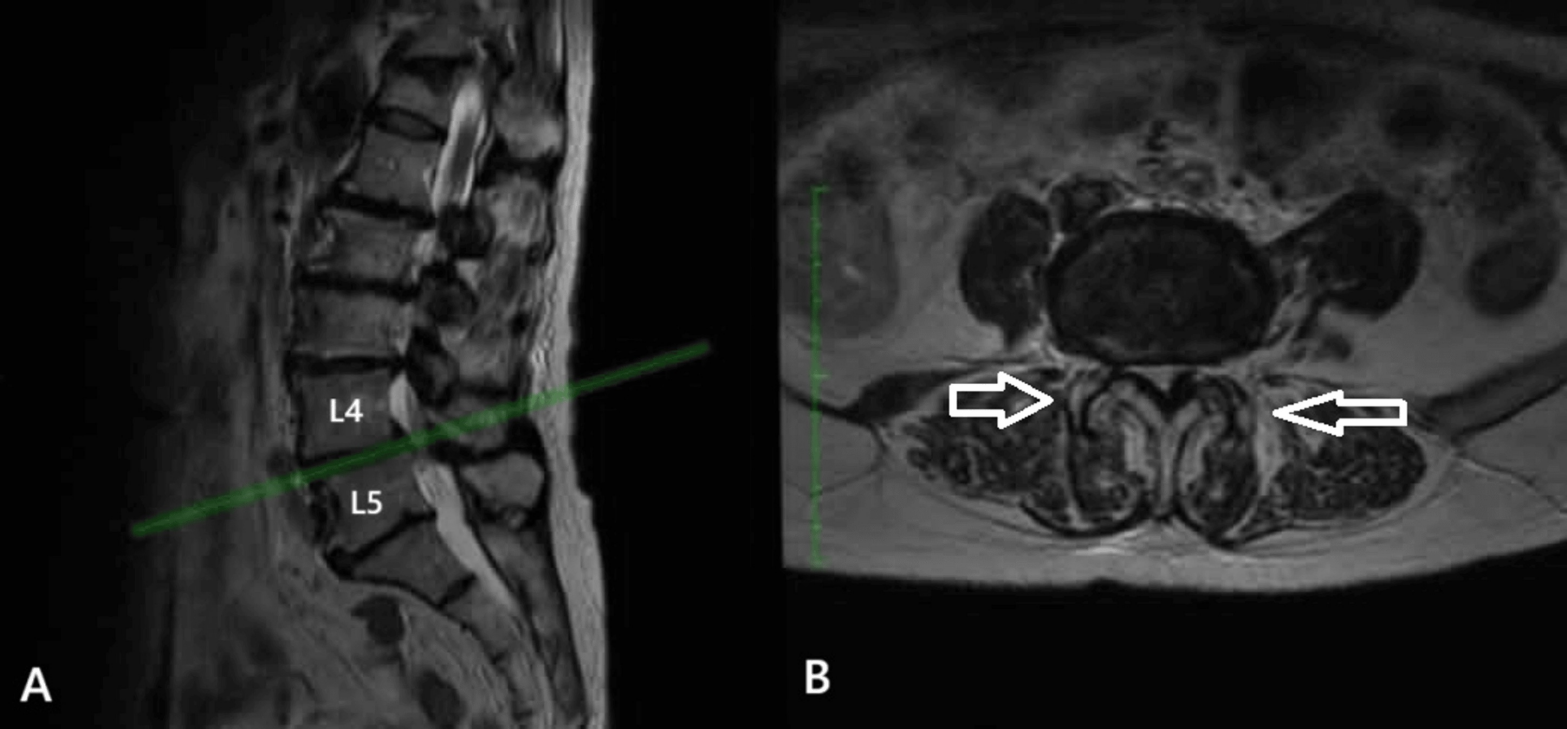
(A) T2-sagittal view and (B) T2-axial MRI views of the lumbar spine in third case. At the L4-L5 level, the arrows with the white outline indicate the bilateral hypertrophic facet joints (grade 4 according to Grogan), with accompanying left facet subluxation.

The third case involved a 75-year-old male with a two-year history of low back pain and progressively worsening bilateral sciatica over the past three months. The patient also reported severe neurogenic claudication, with a walking distance limited to approximately 150 meters. Clinical examination revealed bilateral lower limb muscle strength graded at 4/5, accompanied by hypoesthesia and diminished tendon reflexes in the L4-L5 distribution. Lumbar spine MRI demonstrated central canal stenosis at the L4-L5 level, along with bilateral foraminal stenosis. Additionally, Grade 4 facet joint degeneration according to the Grogan classification was identified at the L4-L5 level, with evidence of subluxation of the left L4-L5 facet joint (Fig. 3). The patient underwent posterior decompression, bilateral foraminotomies, and partial resection (60%) of the affected facet joint, followed by posterolateral instrumented fusion at L4-L5 using transpedicular screws and rods. Postoperatively, the patient experienced immediate symptom relief, was mobilized six hours after surgery, and was discharged the following day with physiotherapy instructions.

Case 4

**Figure 4:**
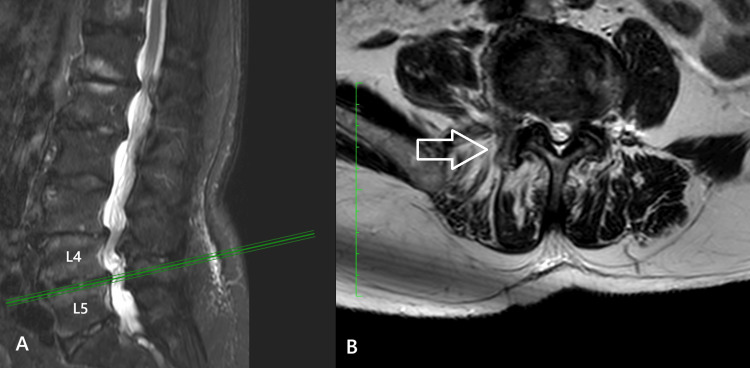
(A) T2-sagittal view and (B) T2-axial MRI views of the lumbar spine in fourth case. At the L4-L5 level, the arrows with the white outline indicate the right facet subluxation.

The fourth case involved a 73-year-old woman with a three-year history of low back pain and progressively worsening bilateral sciatica over the preceding four months. She also reported severe neurogenic claudication, with walking limited to approximately 100 meters. On clinical examination, bilateral lower-limb muscle strength was graded at 4/5, accompanied by hypoesthesia and reduced tendon reflexes in the L4-L5 distribution. Lumbar spine MRI revealed central canal stenosis at L4-L5, together with bilateral foraminal stenosis. In addition, Grade 4 facet joint degeneration, according to the Grogan classification, was noted at L4-L5, with subluxation of the right L4-L5 facet joint (Fig. 4). The patient underwent posterior decompression, bilateral foraminotomies, and partial resection (60%) of the affected facet joint, followed by posterolateral instrumented fusion at L4-L5 using transpedicular screws and rods. Postoperatively, she experienced immediate relief of symptoms, was mobilized six hours after surgery, and was discharged the following day with physiotherapy instructions.

Case 5

**Figure 5:**
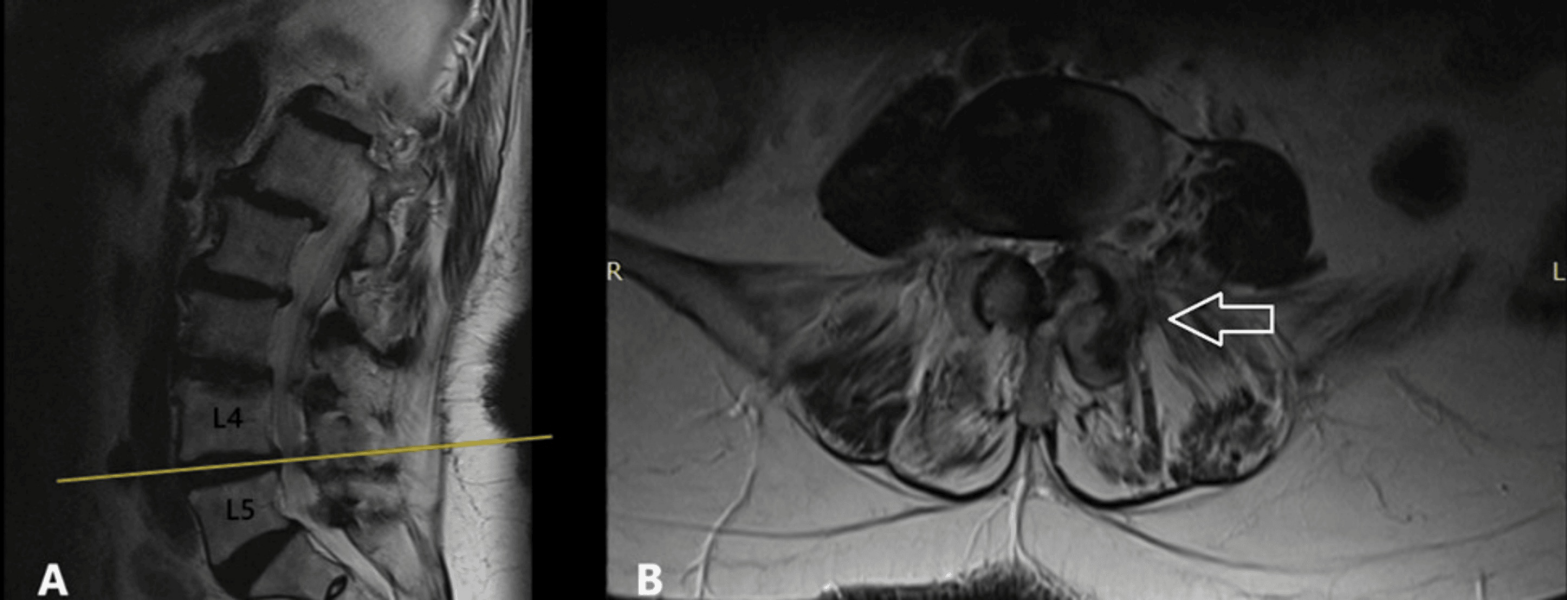
(A) T2-sagittal view and (B) T2-axial MRI views of the lumbar spine in fifth case. At the L4-L5 level, the white arrow indicates left-sided facet joint subluxation and facet hypertrophy.

The last case involved a 72-year-old woman who presented with a six-month history of low back pain and progressive left-sided sciatica, which had significantly worsened over the past two months. Her symptoms persisted despite multiple sessions of physiotherapy and pharmacological pain management. Clinical examination revealed reduced muscle strength in the left lower limb, graded at 3/5, within the L4-L5 dermatomal distribution. Sensory testing indicated hypoesthesia in the same region, and tendon reflexes were diminished in the corresponding distribution. Lumbar spine MRI demonstrated severe left lateral recess stenosis at the L4-L5 level, caused by advanced degenerative unilateral subluxation of the left facet joint (Fig. 5). The facet joint appeared hypertrophic, likely as a result of chronic degenerative changes, contributing to lateral recess narrowing and subsequent neural compression. These facet joint degenerative changes cannot be classified according to the Grogan classification. The patient underwent a left-sided semilaminectomy at the L4-L5 level. To ensure adequate decompression while preserving spinal stability, a foraminotomy was performed with partial resection (approximately 40-50%) of the affected facet joint. Intraoperative assessment revealed no signs of instability before or after the decompression. The patient experienced immediate postoperative relief, was mobilized five hours after surgery, and was discharged 12 hours later. She followed a physiotherapy rehabilitation program.

## Discussion

The cases we have presented represent distinctive examples of degenerative facet joint pathology, uniquely characterized by the presence of facet joint subluxation - a finding not previously described in the literature to date. In these cases, the choice of surgical technique was based on the presence or absence of spinal instability, which, when suspected, was also evaluated intraoperatively. An important consideration in cases where instrumented fusion was performed is the altered anatomy of the facet joints. These changes can significantly affect the anatomical landmarks used for screw placement and thus pose technical challenges for the surgeon. We therefore consider that preoperative identification of facet joint subluxation on MRI plays a critical role: first, by alerting the surgeon to the possible presence of instability requiring intraoperative assessment, and second, by assisting in the planning of an appropriate surgical approach to achieve optimal screw positioning.

The role of hyperextension forces that cause facet dislocations was first described by Watson-Jones and Wilson, thereby laying the fundamental understanding of the biomechanical mechanisms that we still use today [9]. The orientation and biomechanics of lumbar facet joints differ across all vertebral levels, which influences their stability and makes them more susceptible to dislocations in certain areas. The L5-S1 facet joints are the most coronally orientated of all lumbar levels, making them greater at resisting rotational forces and stabilizing the segment against dislocation [5]. On the other hand, the L4-L5 facet joints and laminae are more sagittal oriented, which ultimately allows the joint to be more flexible and extensive, but lacks rotational stability [10,11]. A similar biomechanical pattern to that of the L4-L5 facet joints is also observed at the L3-L4 facet joints. This may also lead to L5-S1 dislocations, whereas the L3-L4 and L4-5 facet joints are able to withstand the same amount of force.

The sagittal plane arrangement makes the facet joints vulnerable to hyperflexion and combined flexion-rotation stresses, even though they provide some resistance to isolated rotational forces in a neutral position [12]. Additionally, the lumbar sagittal plane motion becomes greater from rostral to caudal end, which also supports the increased flexibility of the spine at the L3-L4 and L4-L5 level [5,13]. Regarding flexibility, Marras reported that out of the 16 degrees of motion in the L4-L5 region, 14 degrees are for flexion, which further implicates that flexion is the most dominant movement at this level. In contrast, the L5-S1 region only allocates 10 degrees of motion out of 14 towards flexion [14]. Since the L4-L5 level has the greatest ability to flex, dislocation forces tend to be mostly concentrated at the lumbosacral region [10,15]. The L5-S1 region is more stable due to its fixation to the pelvis through the sacroiliac ligament, which theoretically reduces the likelihood of dislocation at this level [10,16]. However, this doesn’t exclude the fact that the flexibility of the L4-L5 region could also make it more prone to instability or dislocation under unique conditions. All these mentioned biomechanical and anatomical differences account for the extensive literature on dislocations in the lumbosacral region, which emphasizes the rarity of our cases.

The rare occurrence of lumbar facet joint subluxations is due to several anatomical and biomechanical factors that contribute significantly to the stability of the lumbar spine. Additionally, Zoltan et al. reported that the vertical orientation of the facet joints in the lumbar spine also contributes significantly to the rarity of dislocations [17]. This vertical orientation would require more force to dislocate, which is why it is more common to have facet dislocations in the cervical spine, as they are more horizontally aligned. Furthermore, the fact that the vertebral bodies are robust and larger, that the joint facets are reinforced by strong collagenous capsules, and that paraspinal muscles collectively provide resistance against hyperextensional forces, also adds significantly to making lumbar facet joint subluxations a rare occurrence [4,5,12]. These features need a substantial flexion-distraction force to separate and dislocate the facet joints, which is something we didn’t see in our cases, but still managed to happen [4,5,12].

However, Roaf reported that when there is a mild flexion present in the thoracolumbar spine, a rotational force can destabilize the spine and lead to dislocations [16]. These kinds of mechanisms often involve fractures of the articular processes, but cases without fractures have also been documented, which show the complex mechanisms of forces that interplay to overcome lumbar spinal stability [16]. The absence of high-energy forces or fractures in our patients highlights the exceptional nature of these cases, making them a rare and noteworthy contribution to the existing literature.

According to Castillo et al., including their case, there have only been nine reported cases of lumbosacral facet dislocations without associated fractures, even though the lumbosacral region is considered the most stable level of the spine [18]. However, facet dislocations in the non-lumbosacral region are even rarer, with only four reported cases documented in the literature [3,4,12,19]. These cases are summarized in Table 1, emphasizing the rarity of facet joint dislocations in the mid to upper lumbar spine. However, although all these cases are non-lumbosacral and without associated fractures, our present cases distinguish themselves further by their degenerative and non-traumatic nature.

**Table 1:** Summary of all reported cases of facet joint subluxation or dislocation in the lumbar spine without associated fractures, excluding lumbosacral cases. (ALL: Anterior longitudinal ligament, PLL: Posterior longitudinal ligament).

Study (author & year)	Demographics (age/sex)	Mechanism of Injury	Level of Injury	Diagnosis	Etiology	Surgical Procedure
Cho et al., 2018 [3]	35 years old/ Male	Single-vehicle motorcycle accident	L4-L5	Left L4 inferior jumped facet, Grade 1 anterolisthesis, ligamentous injury (ALL and PLL)	Traumatic	Open reduction, bilateral posterior L4-L5 fusion, partial decompression
Younus and Kelly, 2021 [4]	24 years old/ Male	Assault with hammer	L3-L4	Right-sided L3/L4 unifacet dislocation, perched facet (<25% listhesis)	Traumatic	Open reduction, single-level L3/L4 posterolateral instrumented fusion
Im et al., 2012 [12]	37 years old/ Male	Compression by 2,000 kg iron plate	L4-L5	Bilateral L4-L5 facet dislocation, anterior slippage, epidural hematoma	Traumatic	Open reduction, posterior interbody fusion with cage, bilateral pedicle screw fixation, laminectomy (L4), hematoma removal
Schiedo et al., 2017 [19]	5 years old/ Female	High-speed motor vehicle collision	L2-L3	Bilateral L2-L3 facet dislocation, Chance-type flexion-distraction injury, severe kyphosis	Traumatic	L2-L3 laminectomy, bilateral pedicle screw placement, reduction of facet joints, hematoma drainage, deformity correction
Our first case	70 years old/ Female	Non-traumatic	L3- L4	Central canal stenosis. Grade 4 degenerative changes of the facet joints (Grogan classification) L3-L4. Bilateral facet joint subluxation at the L3-L4 level.	Degenerative	Posterior decompression and posterolateral instrumented fusion at L3-L4 using transpedicular screws and rods
Our second case	68 years old/ Female	Non-traumatic	L4- L5	Right-sided lateral recess and foraminal stenosis at the L4-L5 level, with Grade 4 degenerative changes of the facet joint (Grogan classification). The right facet joint showed signs of impending subluxation.	Degenerative	Right-sided semilaminectomy at L4-L5, foraminotomy, and partial (40%) facet joint resection.
Our third case	75 years old/ Male	Non-traumatic	L4- L5	Central canal stenosis at the L4-L5 level and bilateral foraminal stenosis. Grade 4 facet joint degeneration (Grogan classification) at the L4-L5 level, with subluxation of the left L4-L5 facet joint.	Degenerative	Posterior decompression, bilateral foraminotomies, partial resection (60%) of the affected facet joint and posterolateral instrumented fusion at L4-L5 using transpedicular screws and rods.
Our fourth case	73 years old/ Female	Non-traumatic	L4-L5	Central canal stenosis at the L4-L5 level and bilateral foraminal stenosis. Grade 4 facet joint degeneration (Grogan classification) at the L4-L5 level, with subluxation of the right L4-L5 facet joint.	Degenerative	Posterior decompression, bilateral foraminotomies, partial resection (60%) of the affected facet joint and posterolateral instrumented fusion at L4-L5 using transpedicular screws and rods.
Our fifth case	72 years old/ Female	Non-traumatic	L4-L5	Unilateral facet subluxation with severe left-sided lumbar stenosis	Degenerative	Left-sided semilaminectomy and foraminotomy, partial facet joint removal (40-50%)

In Table 1, you can see that our cases include elderly patients, compared to previously reported cases where the oldest patient was 37 years of age [12]. This also reveals the important role of chronic degenerative changes in lumbar facet subluxation. So, it is important to differentiate these kinds of cases between traumatic and degenerative etiologies and to consider age-related structural changes as a contributing factor to these rare entities of facet joint subluxation.

Grogan et al. created an MRI grading system that can be used to assess degenerative changes in facet joints [6]. It classifies the severity and extent of cartilage loss, subchondral bone alterations, and osteophyte formation into four separate grades. However, Grogan’s system does not currently account for cases where degeneration could potentially lead to mechanical instabilities like facet joint subluxation [6,20]. This is especially limiting in cases like ours, where facet joint degeneration is combined with facet subluxation, leading to instability and functional impairment. To address this gap, we propose adding a fifth grade to the Grogan system. This new grade would allow the classification to include joint misalignment or partial dislocation (subluxation) that is visible on MRI scans. Grade 4 only focuses on severe cartilage loss and bone changes, while Grade 5 would encompass the degenerative changes seen in Grade 4, along with the additional presence of facet joint subluxation (unilateral or bilateral). By doing so, the grading system would maintain its focus on MRI-based findings while also acknowledging the functional consequences of degeneration [6,20].

Including this grade would allow clinicians to better categorize cases like ours, and this modification adds value to the existing system by making it more relevant for cases that involve both structural and functional consequences of degeneration.

## Conclusions

Facet joint degenerative changes combined with degenerative facet joint subluxation, as seen in our cases, represent rare clinical entities that can lead to progressive neurological deficits and significant functional impairment. The absence of trauma or fractures in this condition is not adequately addressed by existing classification systems, highlighting the need for a modified system that incorporates non-traumatic and degenerative causes. Understanding facet joint subluxation is important in cases requiring instrumented fusion, as the altered anatomy can create challenges during screw placement. Early diagnosis and appropriate surgical intervention are essential for restoring function and preventing further neurological deterioration.
